# MnO_2_/TiO_2_-Catalyzed ozonolysis: enhancing Pentachlorophenol degradation and understanding intermediates

**DOI:** 10.1186/s13065-024-01194-3

**Published:** 2024-05-09

**Authors:** Cristian Valdés, Cristina Quispe, Rubén A. Fritz, Rodrigo Andler, Jorge Villaseñor, Gina Pecchi, Edgardo Avendaño, Alvaro Delgadillo, William N. Setzer, Javad Sharifi-Rad

**Affiliations:** 1 Centro de investigación de Estudios Avanzados del Maule, Vicerrectoría de Investigación y Postgrado, Universidad Católica del Maule, Avenida San Miguel 3605, Talca, Chile; 2 Facultad de Ciencias de la Salud, Universidad Arturo Prat, Casilla 121, Iquique, 1110939 Chile; 3 Dirección de Investigación Científica y Tecnológica. Vicerrectoría de Investigación, Desarrollo e Innovación, Universidad de Santiago de Chile, Avenida Libertador Bernardo O’Higgins 3363, Santiago, Chile; 4 Escuela de Ingeniería en Biotecnología, Universidad Católica del Maule, Avenida San Miguel 3605, Casilla 617, Talca, Chile; 5 Laboratorio de Fisicoquímica, Instituto de Química y Recursos Naturales, Universidad de Talca, 2 Norte 685, Casilla 721, Talca, Chile; 6 Facultad de Ciencias Químicas, Universidad de Concepción, Edmundo Larenas 129, Concepción, Chile; 7 Departamento de Química e Ingeniería Química, Facultad de Ingeniería, Universidad Nacional Jorge Basadre Grohmann, Avenida Miraflores s/n, Tacna, 23001 Perú; 8 Departamento de Química, Facultad de Ciencias, Universidad de La Serena, Casilla 599, Benavente 980, La Serena, Chile; 9 Department of Chemistry, University of Alabama in Huntsville, Huntsville, AL 35899 USA; 10 Facultad de Medicina, Universidad del Azuay, Cuenca, Ecuador

**Keywords:** Dechloration, Pentachlorophenol, Ozonation, MnO_2_/TiO_2_, Fukui

## Abstract

Pentachlorophenol is a pesticide widely known for its harmful effects on sewage, causing harm to the environment. In previous studies, our group identified adsorption as a crucial factor in catalytic ozonation processes, and subsequent observations revealed the catalyst’s role in reducing toxicity during degradation. In this research, we quantified organochlorine intermediates and low molecular weight organic acids generated under optimal pH conditions (pH 9), with and without the catalyst. Additionally, we assessed the reactivity of these intermediates through theoretical calculations. Our findings indicate that the catalyst reduces the duration of intermediates. Additionally, the presence of CO_2_ suggests enhanced mineralization of pentachlorophenol, a process notably facilitated by the catalyst. Theoretical calculations, such as Fukui analysis, offer insights into potential pathways for the dechlorination of aromatic molecules by radicals like OH, indicating the significance of this pathway.

## Introduction

As a consequence of industrial activities, which are essential in the modern life of developed countries, several environmental hazards have arisen. When developing countries undergo modernization, they encounter similar problems, which may be even more acute due to the lack of supervision, environmental policies, economic resources, and scientific, technological, and human capabilities needed to address them. Water, as one of the most crucial natural resources, has been significantly affected in recent decades [1].

The production and usage of organohalogenated compounds have raised growing concerns regarding their impact on terrestrial and aquatic ecosystems, particularly the toxicological effects they exert.

Pentachlorophenol (PCP) is considered one of the most toxic organochlorines due to its biocidal properties, posing significant risks to various soil and water organisms [2]. Even at low concentrations, such as 1 mg/L, PCP can adversely affect aquatic invertebrates (mollusks, crustaceans) and vertebrates (fish) in acute toxicity tests [3]. Particularly vulnerable are reproduction and juvenile stages, with IC_50_ values dropping as low as 0.01 mg/L for fish larvae. PCP exhibits high toxicity regardless of the route, amount, or frequency of exposure, with mean lethal doses (LD_50_) ranging between 27 and 205 mg/kg of body weight under different conditions [3].

PCP can be absorbed through dermal, oral, and inhalation routes, and despite its toxicity, it does not bioaccumulate in the body. Its elimination half-life in urine is approximately 33 h [4].

Its impact on humans and the environment has been observed over time. Humans are inevitably exposed to PCP through contaminated water and food (Schmied-Tobies et al., 2021). Concentrations of PCP have been reported in spring water ranging from 70 to 140 mg/L, in rivers at 10.5 mg/L, and in industrial effluents at concentrations of 25 to 150 mg/L. Extremely high concentrations of PCP, ranging from 56 to 190 mg/L, are found in groundwater deposits and in the vicinity of sawmills using chlorophenols [5].

The Advanced Oxidation Processes (AOP) may constitute soon one of the most used technological resources in the treatment of contaminated water with dangerous or toxic organic products, which are refractory to the action of conventional biological and chemical treatments of water. These relatively new techniques have been defined by [6] as the processes that involve the generation of oxidizing radicals in sufficient quantity to affect water purification.

AOP usually makes use of the combination of different precursors to generate in situ powerful chemical oxidants such as the hydroxyl radical (HO^•^). These processes are heterogeneous photocatalysis, Fenton, photo-Fenton, catalytic ozonation processes, and treatment of the contaminants with hydrogen peroxide and irradiation with ultraviolet light. The effectiveness of these processes varies depending on the compound being treated [7].

Ozone has many advantageous characteristics in its use; when rapidly decomposing in water, it generates free radicals that provide a high oxidative capacity. This oxidation capability, coupled with hydroxyl radicals, enhances its ability to decompose many organic compounds, including organochlorides. Ozone does not produce secondary pollutants during its degradation [8].

Compared with the homogeneous catalytic ozonation process, the heterogeneous catalytic ozonation has attractive advantages such as reusability, high mineralization degree, and no secondary pollutants.

The interaction of ozone in the catalytic process can be explained according to previous studies by our group, where it was found that a key factor in the degradation process is the adsorption of ozone/organic compound onto the catalyst support. It was found that adsorption and degradation depended on pH [9]. The highest values of adsorption and degradation were reported at pH 2.5 using the MnO_2_/TiO_2_ catalyst. It was reported that the solution pH can significantly affect the decomposition of molecular ozone and, consequently, the generation of hydroxyl radicals, which are essentially the oxidizing agent.

In these processes, the main catalysts are metal oxides (MnO_2_, TiO_2_, Al_2_O_3_) and also metals or metal oxides supported on metal oxides (e.g., Cu/Al_2_O_3_, Cu/TiO_2_, Ru-CeO_2_, VO/TiO_2_, VO/silica gel and TiO_2_/Al_2_O_3_, Fe_2_O_3_/Al_2_O_3_) [4, 7, 9–12]. Various studies suggest that the mechanisms by which ozone is decomposed on the surface of the heterogeneous catalyst depend on the characteristics of the metal oxide, such asst, the pH of the solution that influences the active sites of the surface, and the reactions of decomposition of ozone in aqueous solutions. For the heterogeneous catalytic ozonation, there are three primary reaction mechanisms governing the catalytic ozone decomposition [13] (1) ozone decomposition on oxidized/reduced form of metal oxide or on the surface of the supported metal oxide; (2) decomposition of ozone on Lewis active sites of metal oxides; (3) ozone decomposition on the hydroxyl groups of metal oxides. It may be concluded that all active sites participate to the ozone decomposition [14].

The low solubility and instability of ozone in water leads a problem. Thus, ozonation is carried out with the addition of homogeneous or heterogeneous catalysts, which are called catalytic ozonation processes (COP). COPs have emerged as an efficient treatment method, which improves the removal efficiency of all types of organic pollutants.

Several solid catalysts play an important role in heterogeneous catalytic ozonation. Among them, zeolites, SiO_2_, Al_2_O_3_, TiO_2_, MnO_2_, FeOOH, and bimetallic oxides are studied as possible catalysts [15]. However, different studies have reported that there are different mechanisms of ozonation. The surface properties and the pH of the solution are the factors that influence the properties of the surface-active sites of the catalyst (such as Lewis acid sites and Bronsted acid sites), which have a strong relationship with catalytic efficiency [9, 16, 17].

Our group has worked on the degradation of organic compounds through catalytic ozonation processes. During the degradation process, the presence of catalysts such as ZnO [18], Rh/TiO_2_ [9] and MnO_2_/TiO_2_ (Quispe et al., 2018) demonstrated the generation of different intermediaries and even showing an effect on reducing toxicity.

Within catalytic processes, heterogeneous catalysis has the significant advantage that its catalysts can be reused in multiple cycles, making this process very attractive. In this regard, new catalysts contributing to dehalogenation processes have emerged in recent times. For example, the TiO_2_ –Fe_2_O_3_ type catalyst showed high catalytic activity in the dehalogenation of aromatic halide compounds [19]. The dehalogenation mechanism is related to the excitation of the catalyst by visible light, known as a photo redox catalyst, generating electron transfer that can help generate the alkyl radical, which is neutralized by the abstraction of a hydrogen atom [20].

An experimental and theoretical study of the photochemistry of CHBr_3_ in water reveals dehalogenation and the formation of HCOOH and CO as reaction products. The results of theoretical calculations indicate that the OH insertion/HBr elimination reaction and subsequent reactions lead to the production of final products [21].

Given the various degradation mechanisms that can occur, understanding which intermediates are favored as a result of the degradation of an organochlorine compound is crucial. This study evaluates the reactivity of PCP based on theoretical calculations, its relationship with the primary intermediates generated, and the quantification of these intermediates in relation to the presence of the catalyst.

## Experimental and methods

### Reagents and chemicals

Pentachlorophenol 86% p.a. (Aldrich) was used without further purification, hydrogen peroxide reagent grade 30% (Merck), analytical grade reagents, or calibration standard supplied by Merck or Mallinckrodt were used for HPLC measurements. All solutions used in the dejection studies were prepared using double distilled water. The catalyst was prepared using a commercial TiO_2_ Degussa P25 and Mn(NO_3_)_2_.4H_2_O (Merck).

Analytical grade reagents or calibration standards supplied by Merck or Mallinckrodt were used for HPLC measurements. All solutions used in the dejection studies were prepared using double distilled water.

O_3_ as an oxidizing agent was produced by an OZOCAV generator from pure O_2_, reaching the reactor at a constant flow of 50 mL/min and a concentration of 22.11 ppm, which was measured in line with a GENESYS II spectrophotometer at λ 254 nm [22].

### Catalyst preparation and characterization

Catalyst MnO_2_/TiO_2_ was prepared using TiO_2_ Degussa P25 as support with 1% w/w MnO_2_, by impregnation at 35ºC with an aqueous Mn(NO_3_)_2_ solution using the incipient wetness method. The solid was dried at 120ºC for 12 h and calcined in air at 500ºC for 4 h.25 [11]. Its characterization was by evaluated specific surface area and porosity in an automatic Gemini 2370 Micromeritics system, from the N_2_ adsorption isotherm at -196ºC in the relative pressure range of 0.05–0.995. X-ray analysis for the catalyst was made on a Bruker D8 Advance instrument with Linear LynxEye detector, BraggBrentane geometry, Cu λ = 1.5406 A, 40 KV − 30 mA power, and 0.1 mm fixed optics. The experiments of Temperature Program Reduction (TPR), Temperature Program Desorption (TPD) and the zero point charge (zpc) was previously reported by our group [23].

### Catalytic ozonation system

**Fig. 1:**
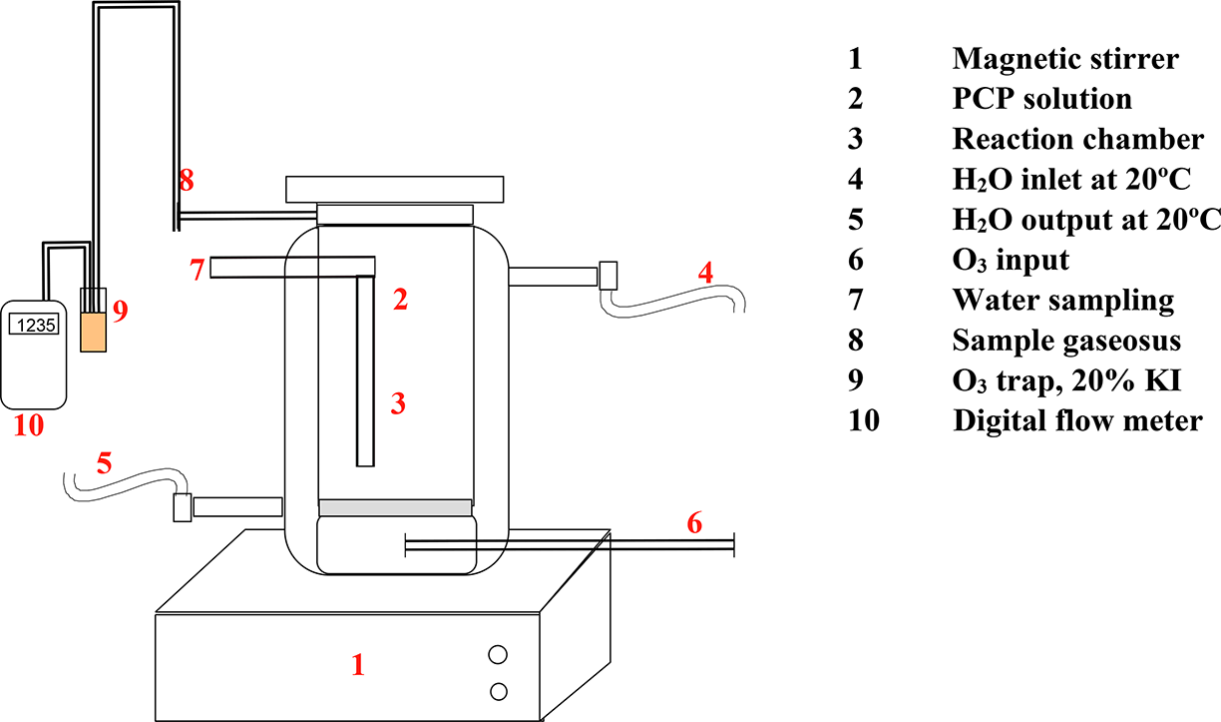
Catalytic system

The heterogeneous catalytic ozonation of pentachlorophenol was performed in a batch reactor, equipped with a double chamber with external circulation, to keep the temperature constant with a sampling system for liquids and gases. The reactions were carried out with O_3_ as an oxidant agent in the reactor with magnetic stirring. In which 90 mL of aqueous PCP solution was placed in each experience. The entry of ozone into the reactor was through a frit placed at the bottom of the reactor (Fig. 1) [24], . The O_3_ was produced by an OZOCAV generator from pure O_2_, reaching the reactor at a constant flow of 50 mL/min and a concentration of 22.11 ppm, which was monitored in line with a GENESYS II spectrophotometer at λ 254 nm [22].

### Analytical methods

Prior to the analysis, the samples were extracted from the reactor at several reaction times (0–60 min) and filtered through a 0.20 mm membrane (Millipore). The concentrations of PCP and its degradation intermediates were measured with an HPLC system coupled to a Perkin Elmer Series 200 chromatograph with a UV–VIS detector. All the intermediates were identified by HPLC by comparing the retention time of the standard solution. The acceptance criterion for the limit of quantification was based in the accuracy for three analyzed samples will present less than 2% variability. The limit of detection was measured taking into account a signal-to-noise ratio of three. The following conditions were used for aromatic compounds; a Merck-Chromolith Performance RP-18 column (4.6 mm×100 mm). The mobile phase was acetonitrile: H_3_PO_4_ (7 × 10^− 3^ mol/L) mixture (40:60), with a flow rate of 1.5 mL/min, and a wavelength detection at 215 nm. The analysis of the acid compounds was made with a Transgenomic ORH-801 column (6.5 mm × 300 mm) with 5 × 10^− 3^ mol/L sulfuric acid as eluent (0.8 mL/min) at 200 nm.

For the determination of CO_2_, a Shimadzu GC-8 A gas chromatograph with a thermal conductivity detector and Porapak-Q column at 50 °C and Helium was used as a carrier at 50 cm^3^/min [24].

### Theoretical study

With the aim of determining the reactivity of pentachlorophenol and intermediaries, local theoretical descriptors in the framework of density functional theory (DFT) were used [25, 26]. The Fukui function (adjusted to radicals) was used according to the following Eqs. [27, 28]:

1 $${f}^{o}\overrightarrow{r }=\frac{\left({\varvec{\rho }}_{N+1}\overrightarrow{\left(r\right)}-{\varvec{\rho }}_{N-1}\overrightarrow{\left(r\right)}\right)}{2}$$

Where ${\rho }_{N+1}(\overrightarrow{r)}$ corresponds to the electron density of the N-electron species when the system gains an electron (anion) and ${\rho }_{N-1}(\overrightarrow{r)}$ when it loses an electron (cation). To obtain the electron density for a given region at the molecule is necessary to divide the molecular space with subsequent integration of the Fukui function in those regions. Then, the Fukui function can be condensed into a specific area of the molecule giving a quantitative indicator of reactivity on that region [29, 30].

In DMol3 [31–33], the condensed Fukui function may be assigned to specific atoms by use of atomic-centered charges such as Hirshfeld [34] scheme. In this way, the electronic density is mapped into a particular atom (k) with charge $\left({q}_{k}\right)$ and the gain $\left({f}_{k}^{+}\right)$or loose $\left({f}_{k}^{-}\right)$ of electron density can be expressed as:

2 $${f}_{k}^{-}={q}_{k}-{q}_{k}^{cation}$$

3 $${f}_{k}^{+}={q}_{k}^{anion}-{q}_{k}$$

And a radical attack is simply represented as the average of those two values.

We used the m-GGA functional M06L with DND basis and implicit solvent (water) using the COSMO methodology available in the software DMol^3^ in every calculation. First, we optimized the geometry of each molecule, followed by a frequency calculation to confirm the structure obtained is indeed a minimum in the potential energy surface. As a final step, condensed Fukui function for a radical attack was estimated and assign to each atom using Hirshfeld charges analysis.

## Results and discussion

**Fig. 2:**
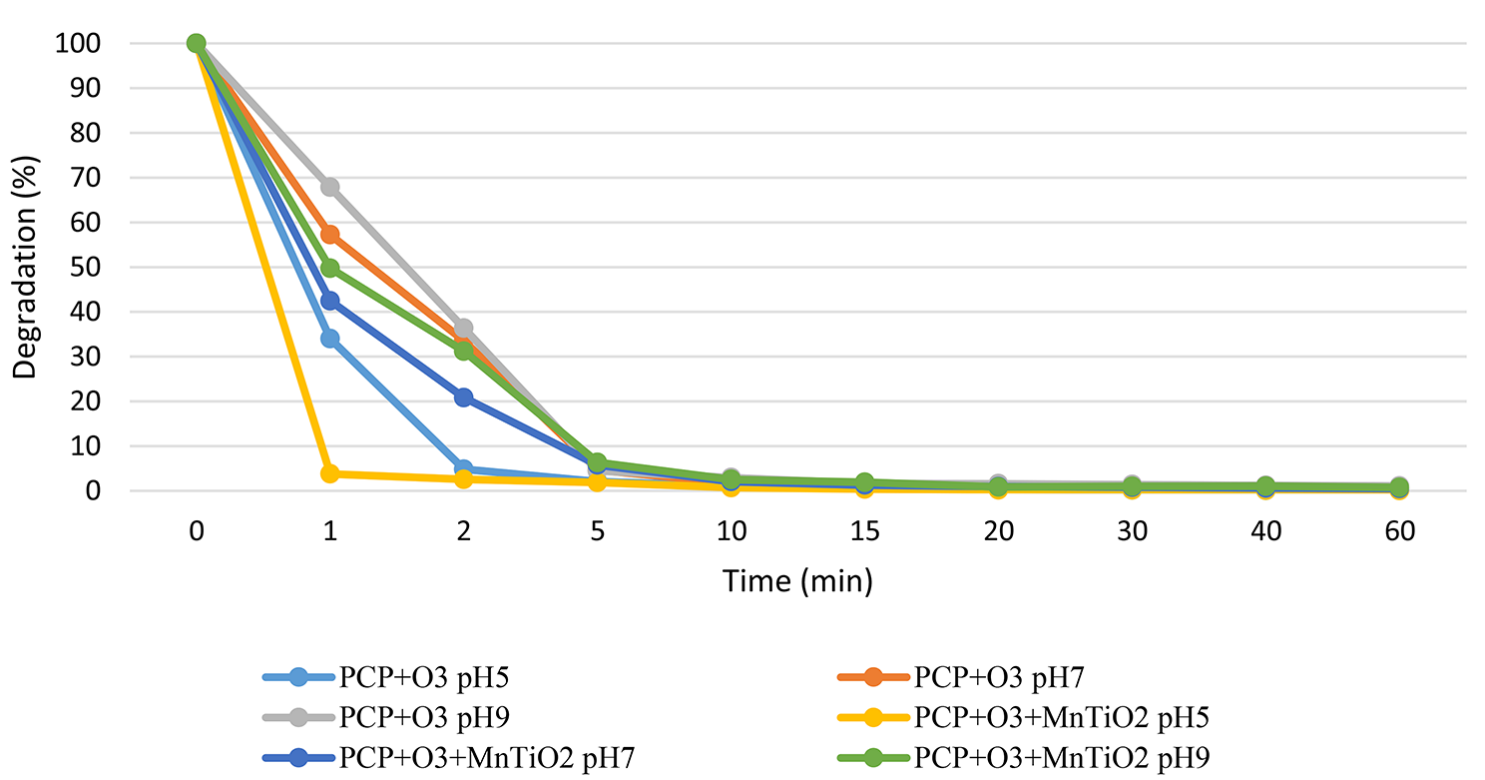
Degradation of pentachlorophenol in presence of ozone

In Fig. 2, the effect of ozone on the degradation of PCP is observed. Initially, the most appropriate reaction conditions regarding pH were established. Experiments were conducted at three different pH levels (acidic pH 5, neutral pH 7, and basic pH 9), using a fixed concentration of O_3_ and controlled temperature. The degradation of pentachlorophenol at different pH levels is depicted in Fig. 2, indicating that the degradation yield at the end of the reaction exceeds 99% within 10 min, regardless of pH. However, differences in the effect of pH on the solution are noticeable before the 5-minute mark of the reaction.

When the same degradation systems are employed in the presence of the catalyst to observe any variation due to heterogeneous catalysis, it is observed that there are no significant differences in the degradation of PCP attributable to the presence of MnO_2_/TiO_2_ (Fig. 2). The use of heterogeneous catalysis does not alter the trend in the percentage of PCP degradation.

After 10 min of degradation, 99% PCP elimination is achieved in both systems, maintaining the same percentage until the end of the catalytic process. Both systems show the highest percentage of PCP degradation at pH 9. However, a noticeable change in the production of intermediates is observed in the presence of the catalyst.

In previous studies conducted by our group, catalytic ozonation of oxalic acid was investigated using MnO_2_/TiO_2_. It was observed that the presence of the metal oxide significantly enhanced the rate of ozonation compared to the non-catalytic reaction. Higher mineralization, as indicated by the evolution of CO_2_, was achieved using MnO_2_/TiO_2_ at pH 2.5 [9]. As will be discussed later, the primary impact of the catalyst is on intermediates.

**Fig. 3:**
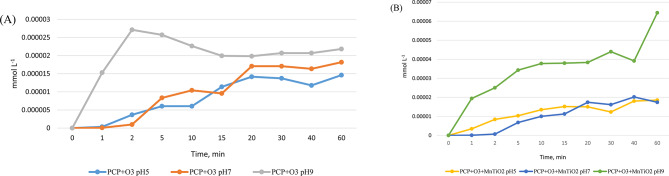
Generation of chlorides in the absence (**A**) and presence (**B**) of the catalyst

Chloride formation is correlated with higher mineralization. When comparing degradation systems at different pH levels in the presence and absence of the catalyst, we observed the highest formation of chlorides at pH 9 (Fig. 3).

The generation of intermediates can be elucidated using theoretical calculations. One suitable tool is Fukui calculations based on radical reactivity. Among the potential transformations is the incorporation of hydroxyl groups into the aromatic molecules presented, wherein the chlorine atoms would be substituted.

**Fig. 4:**
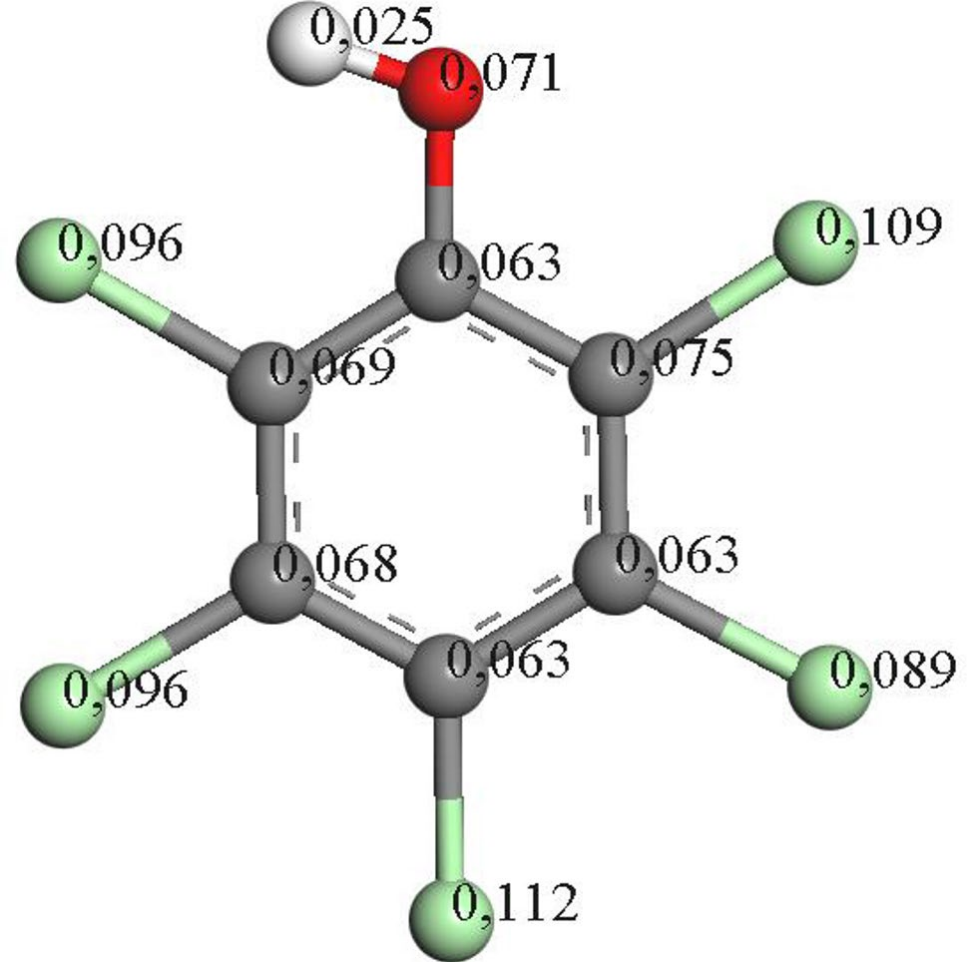
Electronic density distribution of pentachlorophenol by Fukui calculations

The oxidation process of the precursor molecule (pentachlorophenol) involves dechlorination events for the production of the first intermediates. A high susceptibility to radical attack is observed in the chlorinated groups compared to the hydroxyl group of the molecule, with a slight tendency to favor the first attack in the “para” position (Fig. 4).

Among the first intermediaries formed, we find: tetrachloro-1,4-benzoquinone, tetrachloro hidroquinone, tetrachlorocatechol, 2,4-dichlorophenol, each one of the intermediates can continue to have more than one to three dechlorination depending on the case.

In Table 1, the quantification of the main intermediates generated at pH 9 can be observed at different time intervals during the 60-minute reaction, both in the presence and absence of the catalyst. Upon observing the table at the end of the reaction, it is evident that the lowest concentrations of the intermediates found are present in the presence of the catalyst. Conversely, in the initial minutes of the reaction, a higher concentration of intermediates is observed in the presence of the catalyst.

This effect is related to the greater formation of chlorides in the presence of the catalyst at the end of the reaction. This suggests that the presence of the catalyst favors the dechloration of the initial compound and the intermediates that are generated during the reaction.

**Table 1:** Concentration of the main generated intermediates, in the presence and absence of the catalyst

Intermediate	Reaction system									
	PCP + O_3_	PCP + O_3_ + MnO_2_/TiO_2_	PCP + O_3_	PCP + O_3_ + MnO_2_/TiO_2_	PCP + O_3_	PCP + O_3_ + MnO_2_/TiO_2_	PCP + O_3_	PCP + O_3_ + MnO_2_/TiO_2_	PCP + O_3_	PCP + O_3_ + MnO_2_/TiO_2_
	1 min (ppm)		5 min (ppm)		10 min (ppm)		30 min (ppm)		60 min (ppm)	
**2,4-dichlorophenol**	0.0	0.0	0.0	**0.2690** ± 0.0032	0.0	**0.2306** ± 0.0027	0.2000 ± 0.0019	0.1798 ± 0.0021	0.2350 ± 0.0028	0.1970 ± 0.0023
**Tetrachlorocatechol**	0.51679 ± 0.0056	0.4692 ± 0.0351	0.3275 ± 0.2325	0.3984 ± 0.0043	0.0861 ± 0.0013	0.1576 ± 0.0023	0.0536 ± 0.0005	0.0378 ± 0.0005	0.0381 ± 0.0004	0.0313 ± 0.0004
**Tetrachloro-1,4-benzoquinone**	3.068 ± 0.0266	**4.3550** ± 0.0522	1.2980 ± 0.0246	**2.4224** ± 0.046	1.0180 ± 0.193	**1.7470** ± 0.0331	0.0	0.0	0.7000 ± 0.0133	0.0
**Hidroquinone**	0.325 ± 0.0037	**0.95397** ± 0.0147	2.0430 ± 0.0249	**2.6313** ± 0.032	1.9330 ± 0.0318	**2.9198** ± 0.0443	2.7940 ± 0.0480	**2.8664** ± 0.0378	3.3380 ± 0.0473	2.75034 ± 0.0517
**Catechol**	0.974 ± 0.0175	0.9357 ± 0.0130	1.1630 ± 0.0191	0.0	**1.4510** ± 0.0239	0.0	1.3720 ± 0.0253	0.0	1.4540 ± 0.0239	0.0

### Catalyst effect on the evolution of intermediaries

In a prior investigation conducted by our team, we identified the degradation pathway at pH 5 and 9, along with the primary intermediates discovered [12]. From this study, we inferred that the intermediates generated are influenced by the pH of the solution. However, it remained unexplored whether the presence of a catalyst would alter the formation of these intermediates. Therefore, the current study aimed to assess the formation of the main intermediates in the presence of the catalyst, as well as their quantification, to examine the catalyst’s impact at pH 9, along with conducting theoretical calculations on the primary intermediates.

Under the specified conditions, various intermediates are detected, generally appearing and degrading rapidly. In Table 1, Tetrachloro-1,4-benzoquinone (TTC-1,4-BQ) can be observed, reaching a concentration of 4.35 ppm at minute 1 in the presence of the metal oxide catalyst. However, after 10 min of reaction time, its concentration falls below 2 ppm, and it is not detected at the end of the reaction.

TTC-1,4-BQ is a known byproduct generated through the oxidation of pentachlorophenol. Previous studies have investigated its degradation in aqueous systems in the presence of hydrogen peroxide [35]. This compound can undergo oxidation to trichloroacetic acid, as suggested in our previous work [23]. Intermediate TTC-1,4-BQ exhibits a peak at the reaction’s onset, gradually decreasing to basal levels within 20 min. In the absence of a catalyst, this intermediate tends to maintain a concentration of around 1 ppm throughout the reaction time.

We need to elucidate the pathway through which TTC-1,4-BQ is formed from PCP. PCP undergoes dechlorination at the *para* position, where a chlorine atom is substituted with an OH group in the presence of radicals. This OH group, along with the one in the ipso position, favors the keto state at basic pH, leading to the formation of TTC-1,4-BQ.

**Fig. 5:**
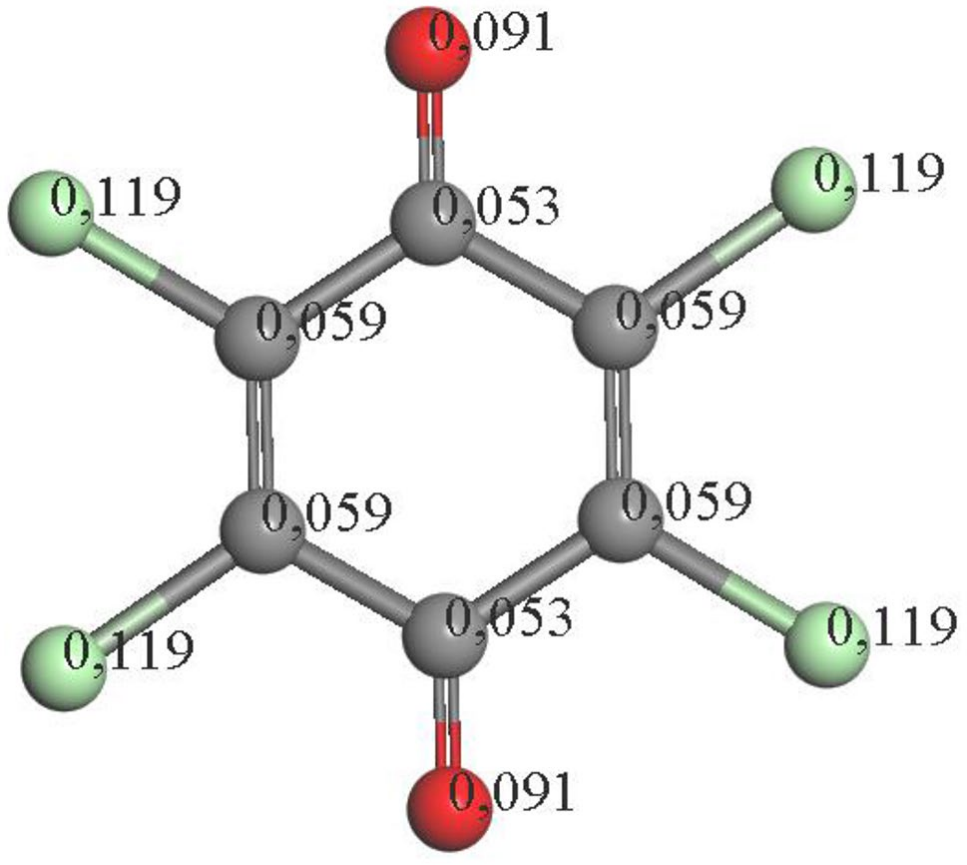
TTC-1,4-BQ electron density distribution using Fukui calculations

Theoretical reactivity studies conducted on the PCP molecule suggest that it undergoes dechlorination by radicals, which attack the chlorine atoms via a two-step mechanism involving the substitution of chlorine by OH, with a preference for the *para* position (refer to Fig. 5).

Upon analyzing the reactivity of PCP, it is observed that the chlorines with the highest electron density are situated at positions 2 and 4, precisely the ones eliminated to generate intermediates in the reaction.

**Fig. 6:**
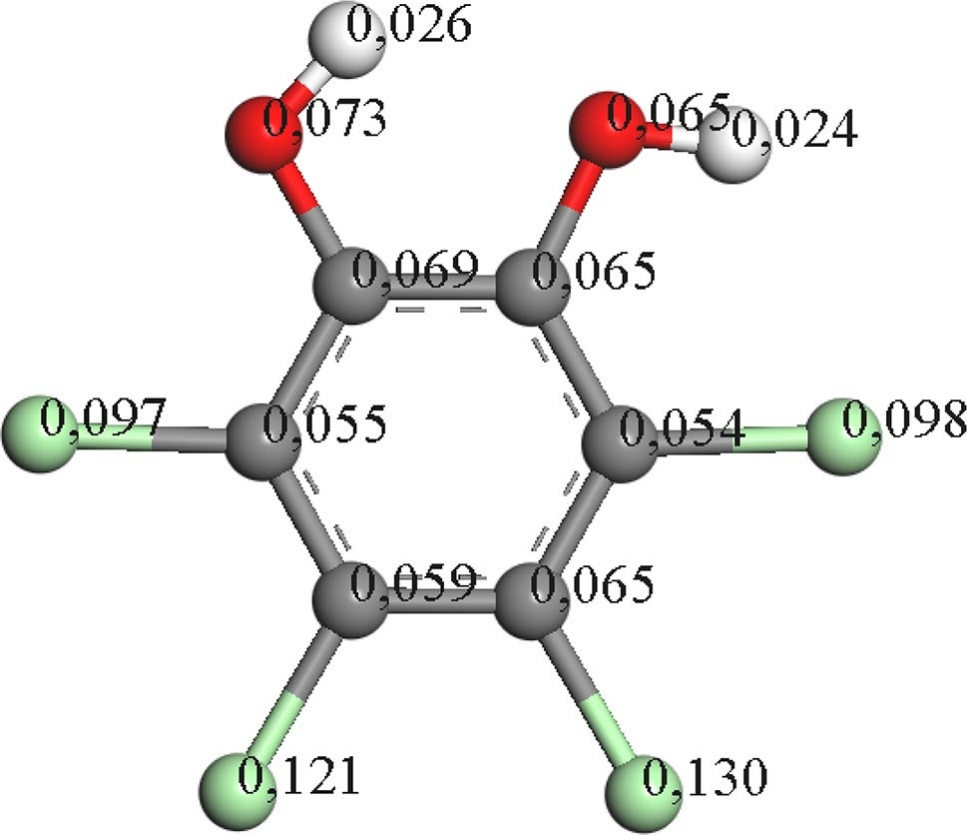
TTC electron density distribution by Fukui calculations

PCP can undergo dechlorination at positions other than the *para* position, such as carbon two, leading to the formation of tetrachlorocatechol (TTC). In Table 1, TTC is shown to initially reach concentrations of 0.7 ppm, decreasing to levels below 0.1 ppm after 10 minutes. However, in the presence of a catalyst, the maximum TTC formation is approximately half, with a higher rate of decay compared to when the catalyst is absent. It’s worth noting that TTC is an intermediate described in the chlorinated residues of the ‘Kraft pulp’ process [36]. TTC is found in some soils and can undergo degradation through oxidative processes [37] and bacterial biodegradation, where mechanisms involving the substitution of Cl groups for hydroxyl have been proposed [38]. According to calculations by Fukui, TTC is dechlorinated via radical attack, potentially leading to the substitution of Cl by OH (see Fig. 6). Complete dechlorination of TTC yields the hydroquinone intermediate, as detected in this study.

**Fig. 7:**
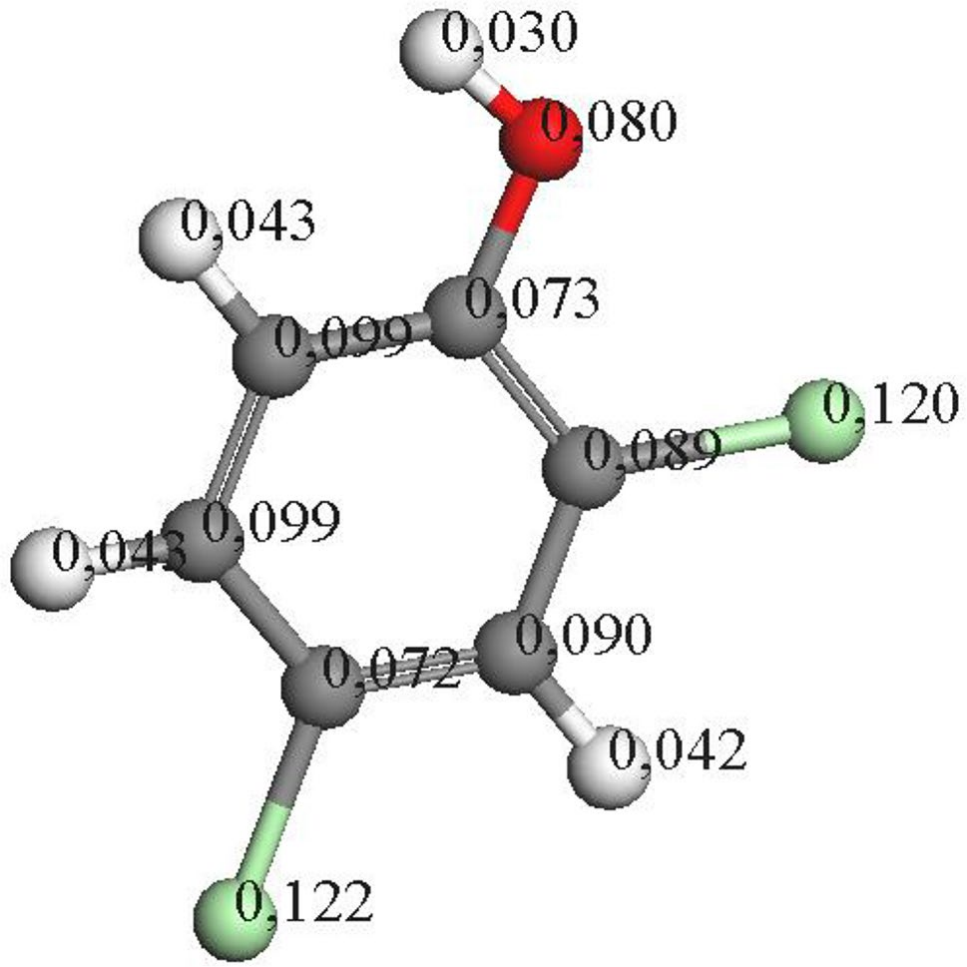
Distribution of electron density of 2,4-dichlorophenol by Fukui calculations

In addition to tetrachlorocatechol, another significant intermediate formed during the dechlorination process is 2,4-dichlorophenol. Table 1 presents the quantification of 2,4-dichlorophenol, highlighting the importance of catalyst utilization, which notably enhances the degradation of these intermediates. Alongside the degradation of chlorinated compounds, oxidation reactions occur, resulting in a mixture of intermediates. The catalyst plays a crucial role in facilitating the degradation of these intermediates. 2,4-dichlorophenol can undergo breakdown in water through radical reactions, with radicals potentially supplied by a combination of ozone and catalysts. This process favors the breakdown of the aromatic ring, ultimately yielding organic acids [39]. Moreover, the Fukui index suggests that dechlorination at the *para* position is more favorable (see Fig. 7), leading to the formation of intermediates with fewer chlorinated groups. Subsequent radical attacks can then facilitate the removal of remaining chlorinated groups.

After complete dechlorination accompanied by an oxidation process, the formation of hydroquinone and catechol compounds is observed. Analysis of Table 1 reveals that in the presence of the catalyst, there is a notable increase in hydroquinone formation initially, with concentrations oscillating around 2.9 ppm from 10 to 30 min of reaction. However, in the system where the catalyst is present, the concentration of hydroquinone decreases, reaching approximately 2.7 ppm within 60 min. Various degradation systems, including Fenton-type, photocatalytic, and sonocatalytic methods, have been reported for hydroquinone degradation as industrial waste, involving the generation of hydroxyl radicals [40].

**Fig. 8:**
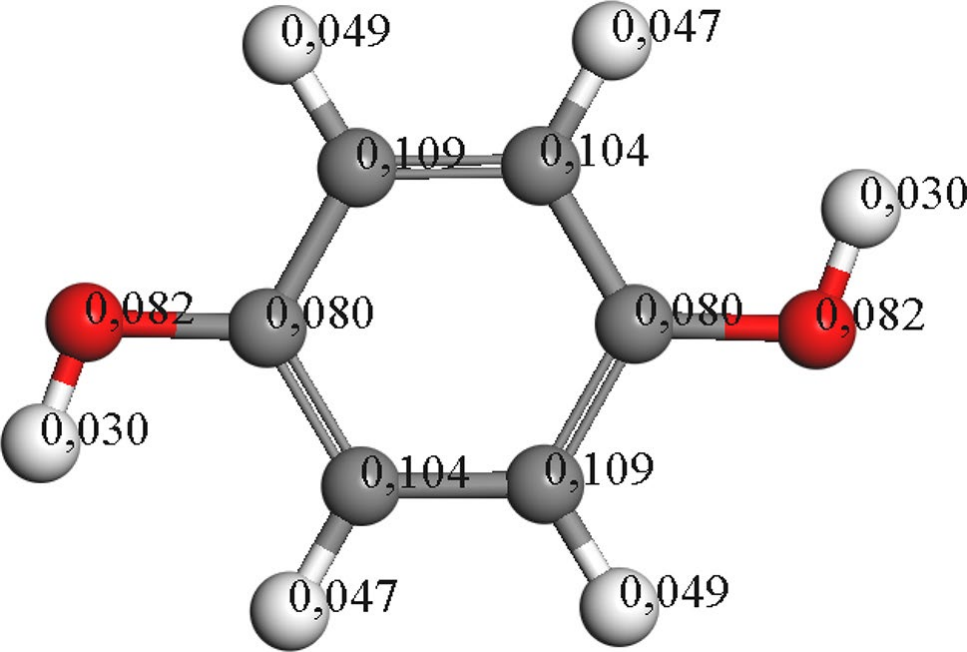
Hydroquinone electronic density distribution by Fukui calculations

Hydroquinone, lacking chlorine in its structure, undergoes oxidation with the promotion of hydroxyl group incorporation. The Fukui calculation depicted in Fig. 8 indicates that radical attacks are favored in carbon groups not anchored to hydroxyl groups, resulting in the formation of organic acids upon ring cleavage.

**Fig. 9:**
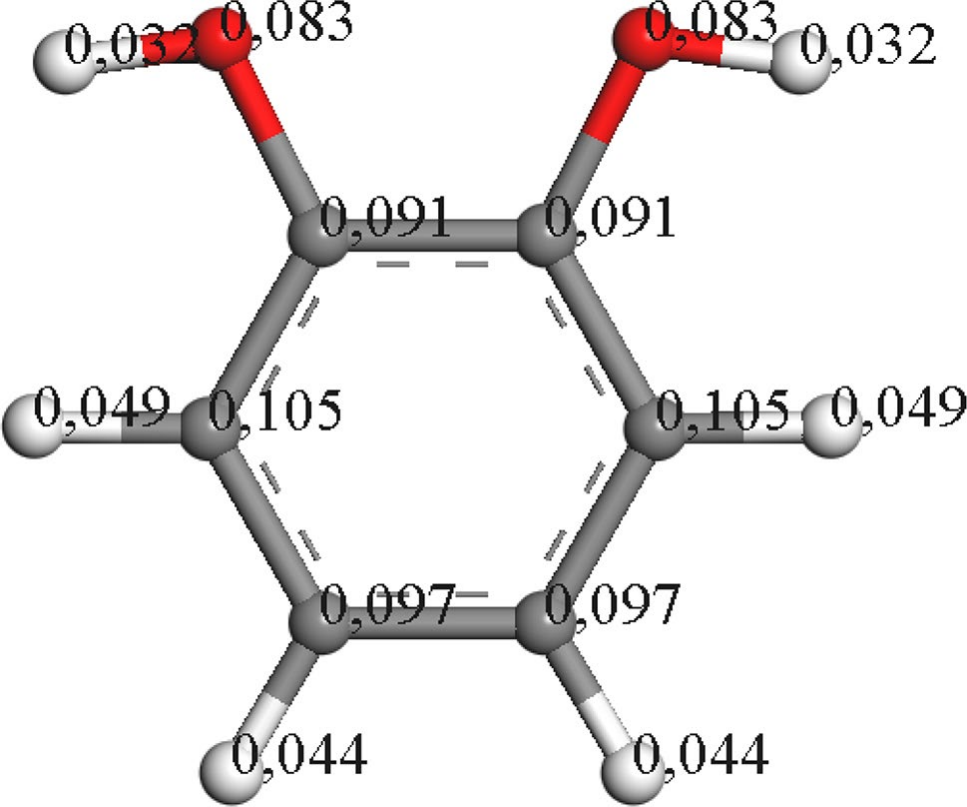
Distribution of catechol electron density by Fukui calculations

Another intermediate formed in the process is catechol, which can result from the dechlorination of 2,3,5,6-tetrachlorocatechol (see Fig. 9). The concentration of catechol formed in the presence and absence of the catalyst is presented in Table 1. Catechol, also known as pyrocatechol, is identified as the most toxic compound detected, with an ecotoxicity value of EC_50_ = 1.66 mg/L in *Daphnia magna*. In the absence of the catalyst, the concentration of catechol reached approximately 1.5 ppm and remained constant until the end of the reaction. Conversely, in the presence of the catalyst, concentrations around 0.9 ppm were detected after 1 min of reaction, followed by an abrupt disintegration, reaching undetectable levels by the end of the reaction.

The presence of catechol has been described as an intermediate in the degradation of phenolic compounds [41]. It is a significant compound in assessing ecotoxicity when precursor molecules decompose, as it is a known industrial waste seeking degradation [42]. Oxidation mechanisms of catechol, involving ozone and radicals such as hydroxyl, have been proposed, with the incorporation of hydroxyl groups into the molecule being a key step [43]. The oxidation of catechol can yield molecules such as pyrogallol or organic acids, as detected in the present study.

### Acid intermediates

**Fig. 10:**
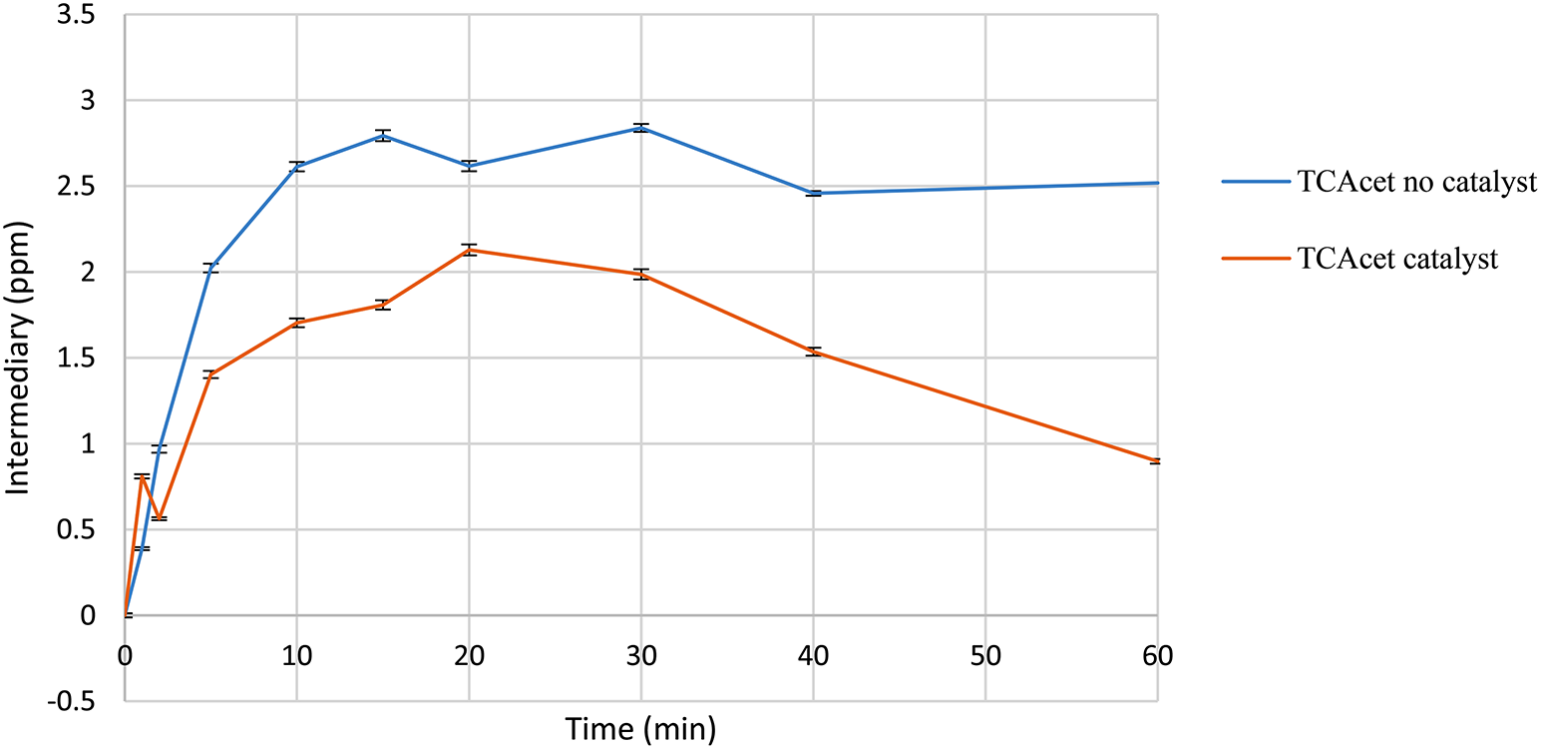
Trichloroacetic acid formation in the presence of ozone and catalyst

The degradation process results in the production of various low molecular weight organic acids. Among the aromatic intermediates detected, trichloroacetic acid, malonic acid, formic acid, oxalic acid, and maleic acid were identified. Trichloroacetic acid (see Fig. 10) was quantified, reaching final concentrations below 1 ppm in the presence of the catalyst, with a noticeable decreasing trend in concentration over time. Studies have suggested that in aqueous systems, photolysis (UV light) and sonolysis (ultrasound) systems can be employed for trichloroacetic acid degradation, with dechlorination being more efficient at higher pH levels [44].

These findings highlight the diverse range of organic acids generated during the degradation process and underscore the potential for various degradation mechanisms to be employed for their removal.

**Fig. 11:**
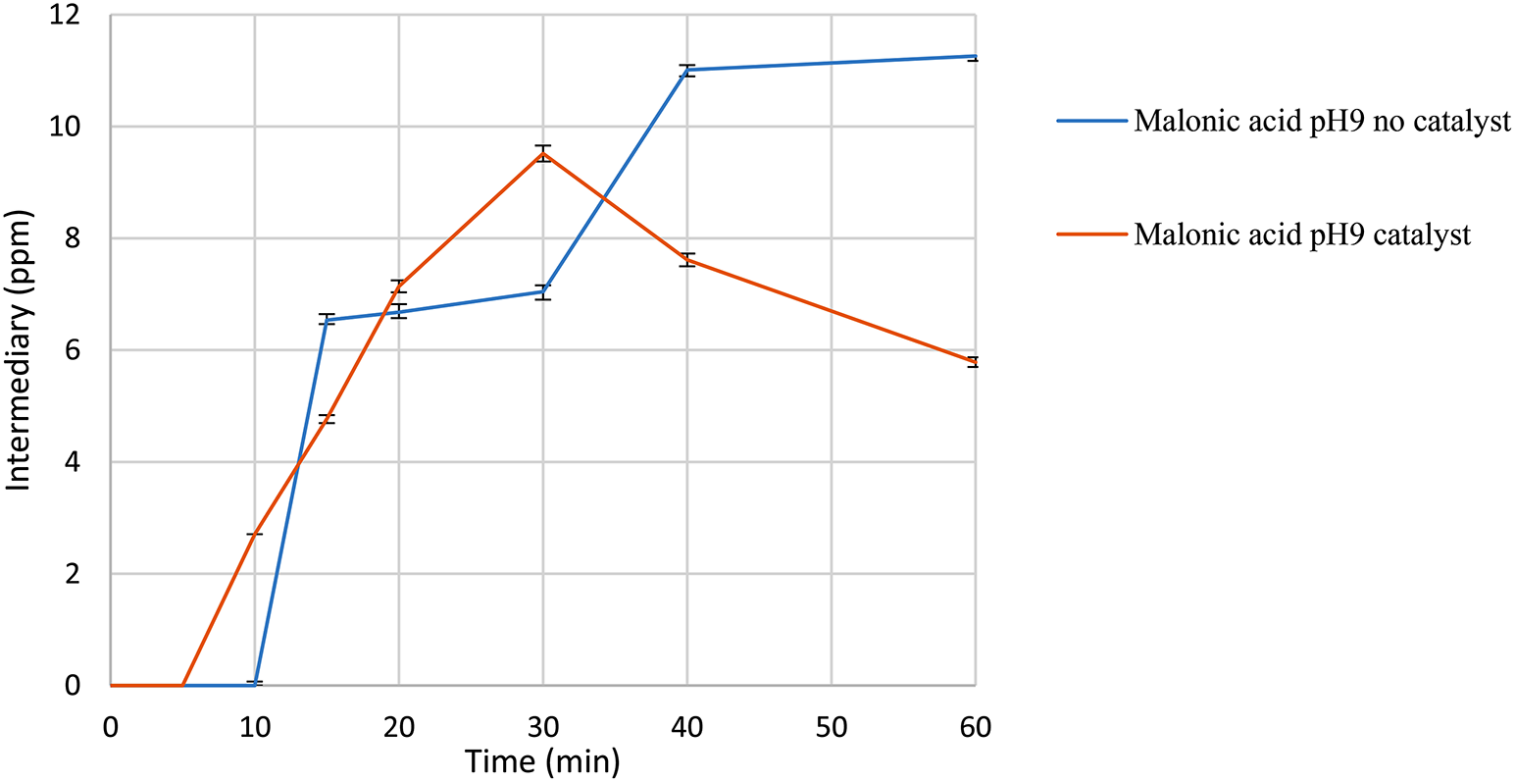
Malonic acid formation in the presence of ozone and catalyst

Malonic acid (see Fig. 11) was identified as one of the organic acids present, exhibiting distinct behavior in the absence and presence of a catalyst. In the absence of a catalyst, malonic acid shows a constant increase, reaching nearly 12 ppm within 60 min. Conversely, in the presence of the catalyst, a peak concentration of almost 10 ppm is observed after 30 min, followed by a gradual decrease to 6 ppm by the end of the reaction. Previous studies have reported the oxidation of malonic acid using ceric ions and radical oxidation in the presence of dissolved oxygen [45].

It is noteworthy that the catalytic system described in this study promotes the generation of oxygen in the aqueous medium, potentially facilitating the oxidation of malonic acid through similar routes. Additionally, the ability to oxidize malonic acid using Mn^+ 3^ ions has been documented, suggesting the possible formation of this ionic species from the catalyst utilized in this work (citation needed). Another alternative is the transformation of malonic acid into glyoxylic acid in the presence of MnO_2_ and HClO_4_, with this catalytic system operating independently of the pH of the medium [46].

**Fig. 12:**
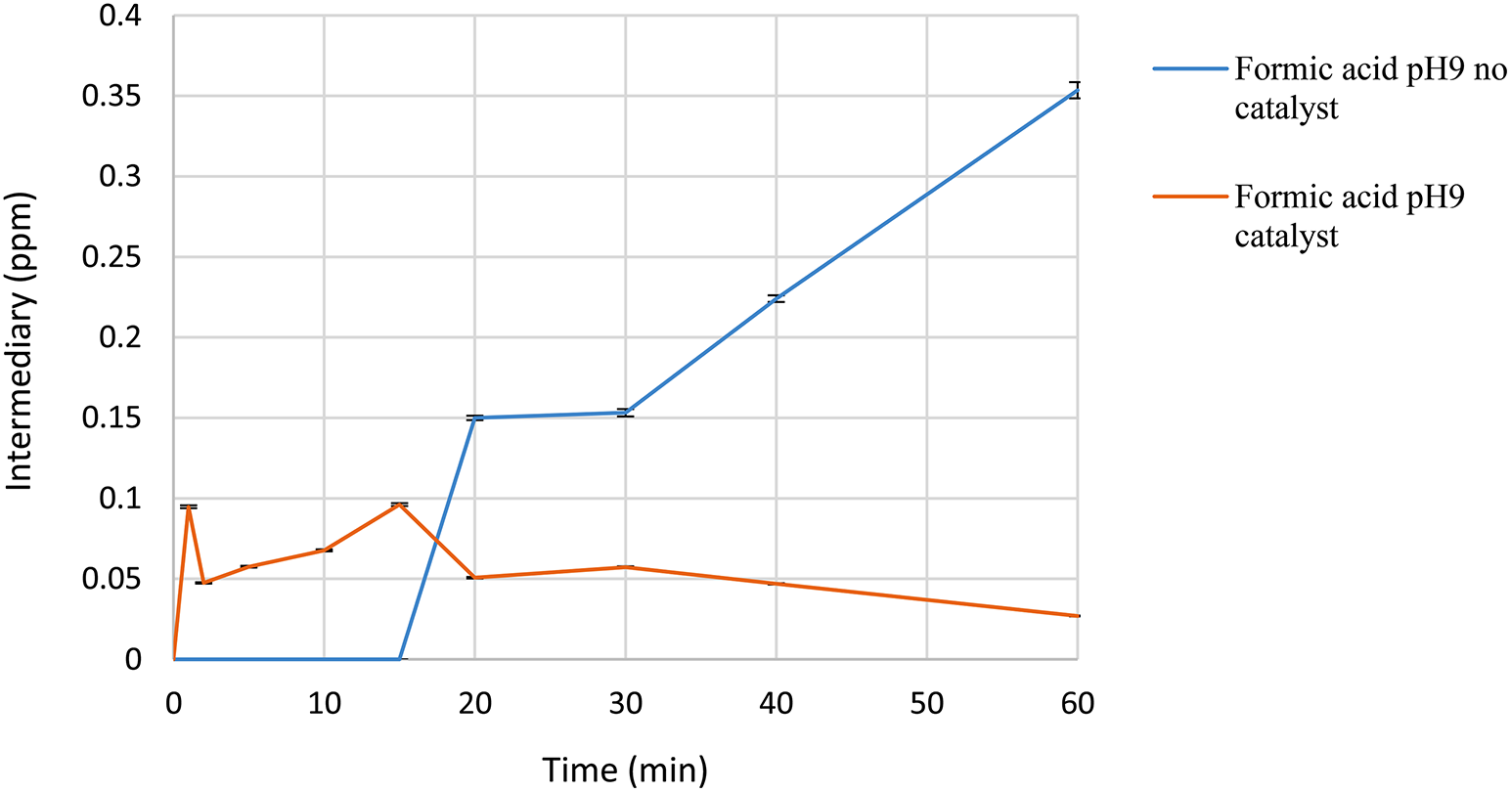
Formic acid formation in the presence of ozone and catalyst

The presence of formic acid was also detected (Fig. 12). It can be observed that in the absence of catalyst, there is a consistent increase in the concentration of this acid, reaching values of 0.35 ppm. However, in the presence of the catalyst, there is a tendency for the concentration not to exceed 0.1 ppm over the course of a 60-minute reaction. Formic acid is a molecule with high energy density, making it compatible with applications as a cell fuel. Its oxidation has been documented in electrochemical processes [47].

**Fig. 13:**
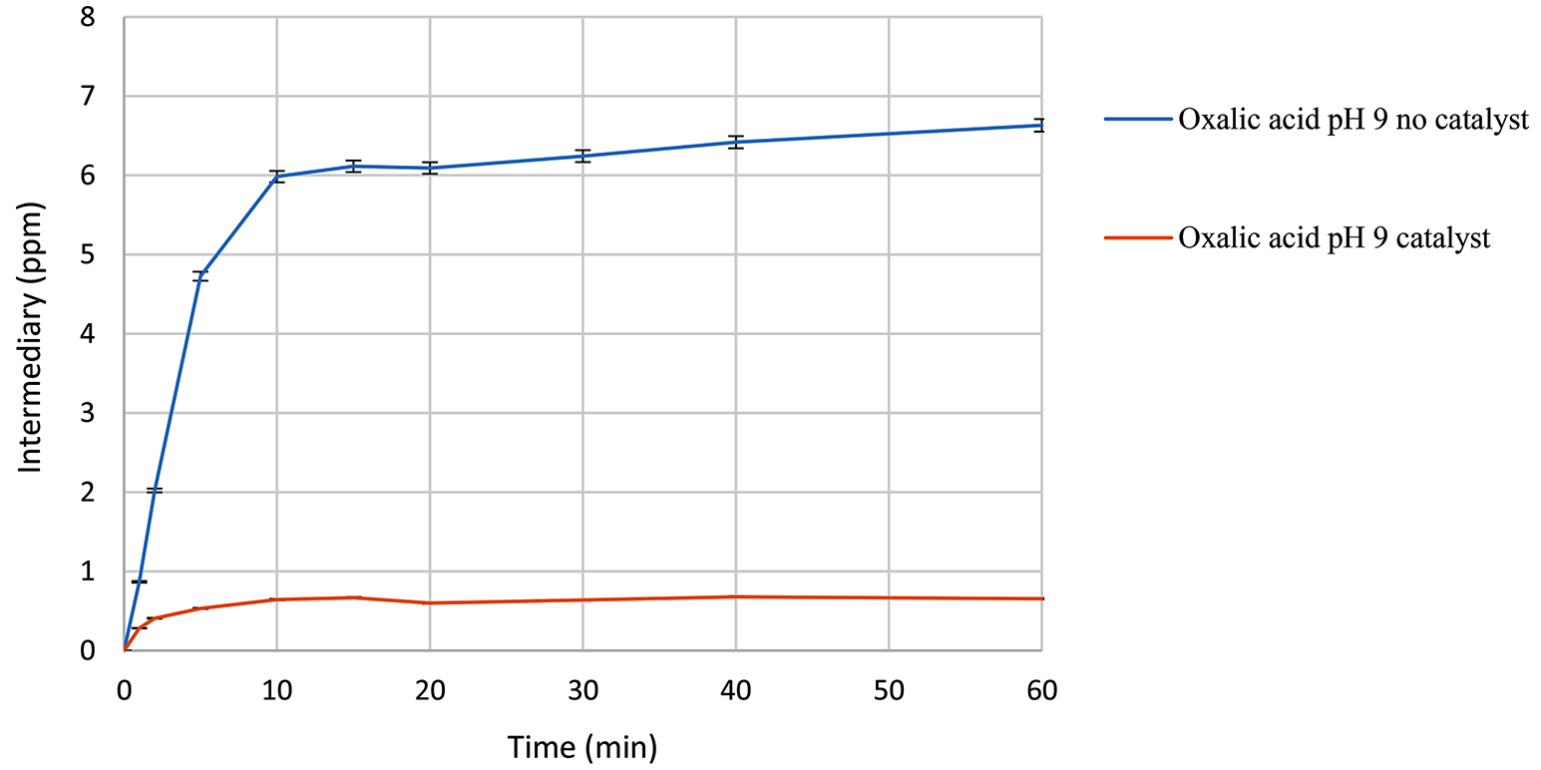
Oxalic acid formation in the presence of ozone and catalyst

The disparities between the conditions with and without the catalyst are evident in the generation of oxalic acid (Fig. 13). In the absence of the catalyst, oxalic acid levels reach 6 ppm, exhibiting a tendency to increase over time. Conversely, in the presence of the catalyst, oxalic acid levels are observed to be less than 1 ppm, with a consistent trend towards these values.

**Fig. 14:**
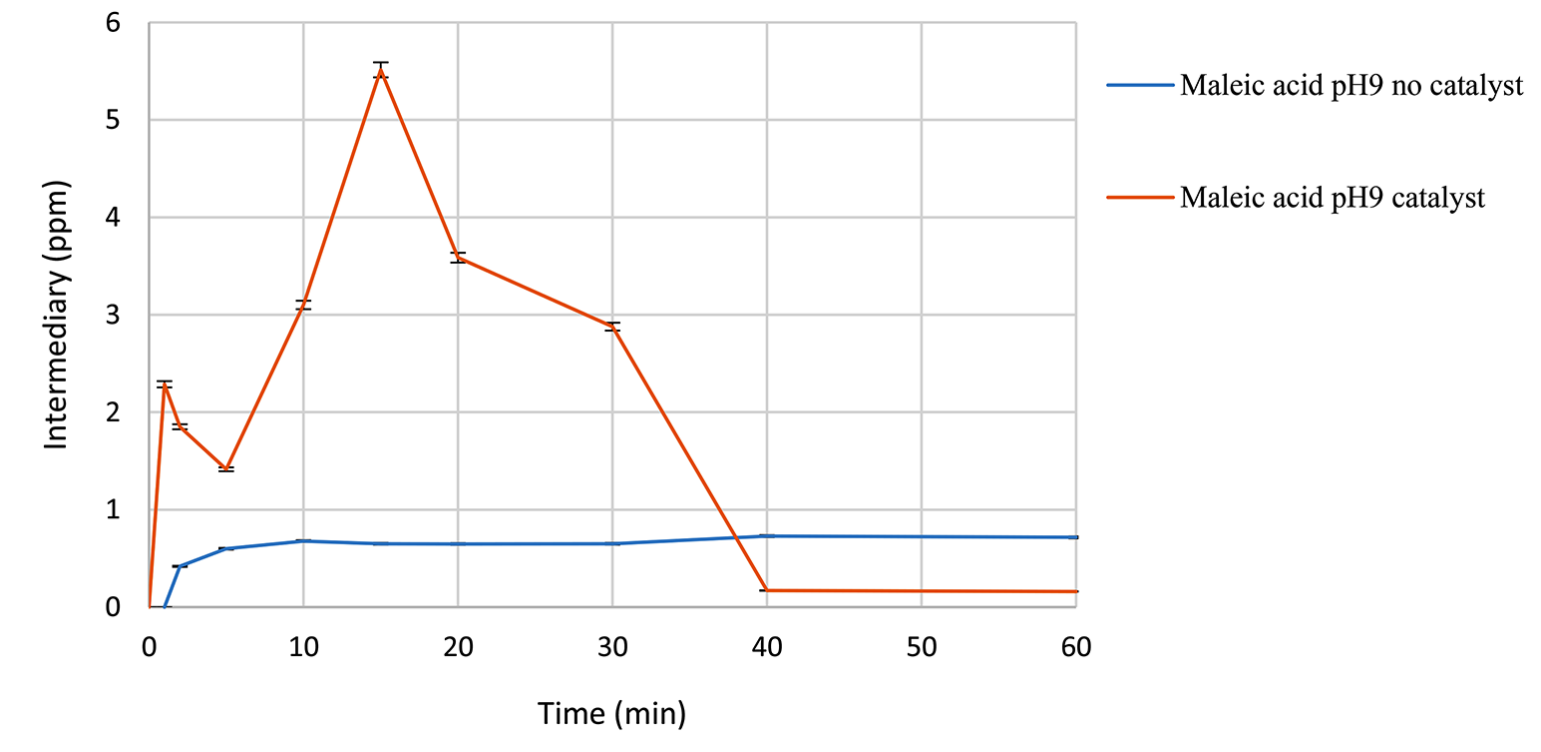
Maleic acid formation in the presence of ozone and catalyst

The quantity of maleic acid detected (Fig. 14) in the presence of the catalyst after 15 min of reaction is higher compared to the reaction without the catalyst. However, it experiences a sudden decrease, dropping to levels below 0.5 ppm, whereas in the absence of the catalyst, it maintains a consistent trend around 0.8 ppm. In the presence of the catalyst, more of this intermediate is generated, yet it also degrades rapidly due to the catalytic system, resulting in lower levels compared to those observed in the absence of the catalyst.

**Fig. 15:**
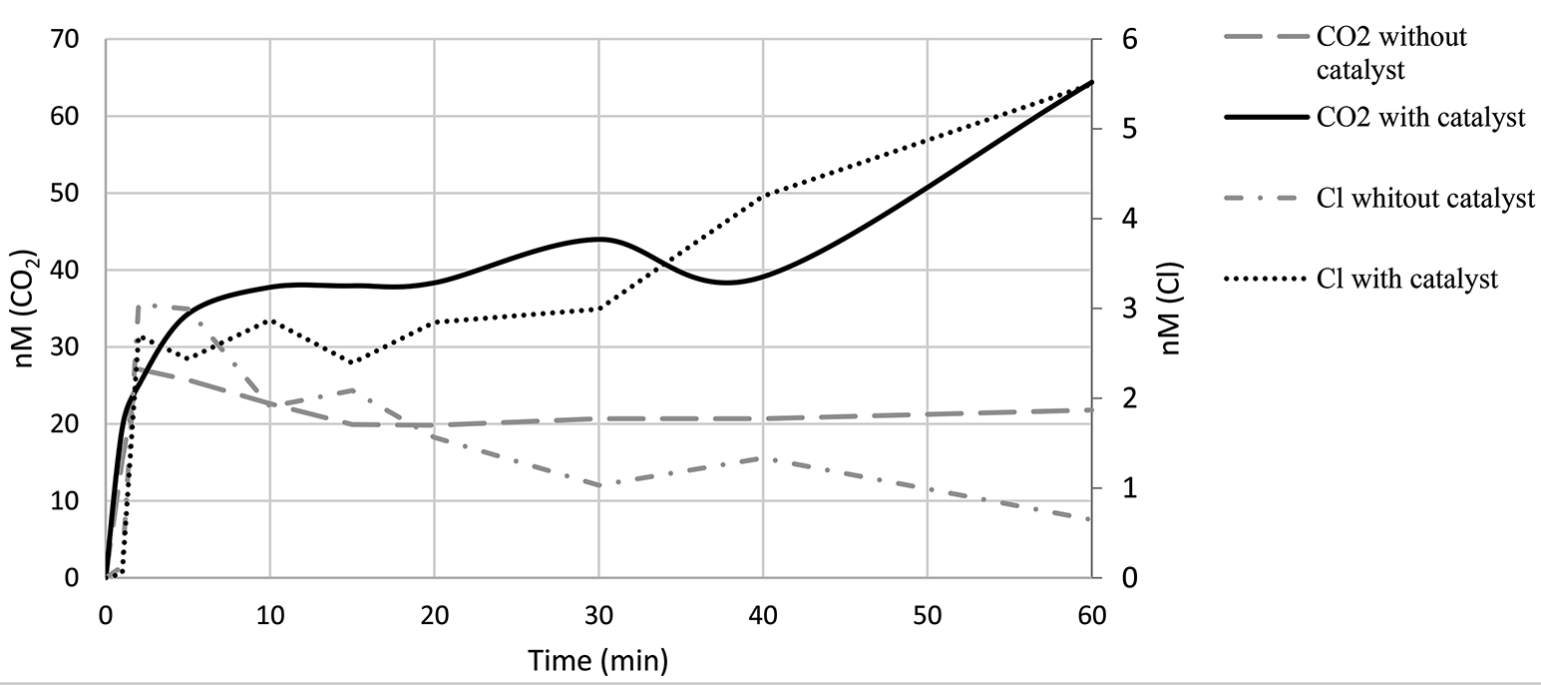
Production of chlorides and accumulation of CO_2_ in the presence and absence catalyst’s

In Fig. 15, the production of chlorides and the accumulation of CO_2_ in the presence of the catalyst are evident. This figure illustrates how the use of the catalyst enables greater dehalogenation and higher mineralization of the starting compound.

As depicted in Fig. 15, the formation of chlorides remains constant throughout the entire reaction period, with a measured concentration of 6.4 × 10^− 5^ ppm at the end of the reaction. The chloride concentration was determined using a calibration curve encompassing six Cl^−^ levels ranging from 10^− 6^ M to 10^− 5^ M, employing a chloride-specific electrode alongside an Orion model 330 potentiometer. Concurrently with the formation of chlorides, the generation of CO_2_ also exhibits an increasing trend in the presence of the catalyst.

Pentachlorophenol mineralization has been previously achieved through biological methods employing microorganisms such as Mycobacterium chlorophenolicum and Sphingomonas chlorophenolica. However, these methods are complex and time-consuming, often taking months for treatment [48]. Additionally, there are systems based on the utilization of biofilms that yield satisfactory results, albeit they rely on potentially hazardous reagents such as H_2_ [49].

## Conclusions

The behavior of intermediates during the degradation of pentachlorophenol exhibits variability, as their disappearance can occur through a multitude of pathways. Our findings suggest that the presence of a catalyst leads to a shorter duration of intermediates over time. The appearance of CO_2_ indicates a correlation between the disappearance of pentachlorophenol and its mineralization, a process significantly enhanced in the presence of the catalyst.

The dechlorination process can be facilitated by advanced oxidation processes (AOPs), involving the attack of hydroxyl radicals that replace chlorine atoms in the aromatic ring. Theoretical calculations, such as Fukui’s analysis, provide insight into feasible dechlorination pathways of aromatic molecules by radicals like OH, suggesting this route as particularly significant.

## Data Availability

Data will be made avaiable on reques.

## Abbreviations

AOP: Advanced Oxidation Processes
COP: catalytic ozonation processes
DFT: density functional theory
EC_50_: half maximal effective concentration
HO^•^: hydroxyl radical
HPLC: high performance liquid chromatography
IC_50_: Half-maximal inhibitory concentration
LD_50_: The mean lethal dose
PCP: pentachlorophenol
TCAcet: Trichloroacetic acid
TTC-1,4-BQ: Tetrachloro-1,4-benzoquinone
TTC: tetrachlorocatechol
